# Bioconversion of Airborne Methylamine by Immobilized Recombinant Amine Oxidase from the Thermotolerant Yeast *Hansenula polymorpha*

**DOI:** 10.1155/2014/898323

**Published:** 2014-01-29

**Authors:** Sasi Sigawi, Marina Nisnevitch, Oksana Zakalska, Andriy Zakalskiy, Yeshayahu Nitzan, Mykhailo Gonchar

**Affiliations:** ^1^Department of Chemical Engineering and Biotechnology, Ariel University, 40700 Ariel, Israel; ^2^The Mina and Everard Goodman Faculty of Life Sciences, Bar-Ilan University, 52900 Ramat Gan, Israel; ^3^Department of Analytical Biotechnology, Institute of Cell Biology, National Academy of Science of Ukraine, Drahomanov Street 14/16, Lviv 79005, Ukraine; ^4^Institute of Applied Biotechnology and Basic Sciences, University of Rzeszow, Sokolowska Street 26, 36-100 Kolbuszowa, Poland

## Abstract

Aliphatic amines, including methylamine, are air-pollutants, due to their intensive use in industry and the natural degradation of proteins, amino acids, and other nitrogen-containing compounds in biological samples. It is necessary to develop systems for removal of methylamine from the air, since airborne methylamine has a negative effect on human health. The primary amine oxidase (primary amine : oxygen oxidoreductase (deaminating) or amine oxidase, AMO; EC 1.4.3.21), a copper-containing enzyme from the thermotolerant yeast *Hansenula polymorpha* which was overexpressed in baker's yeast *Saccharomyces cerevisiae*, was tested for its ability to oxidize airborne methylamine. A continuous fluidized bed bioreactor (CFBR) was designed to enable bioconversion of airborne methylamine by AMO immobilized in calcium alginate (CA) beads. The results demonstrated that the bioreactor with immobilized AMO eliminates nearly 97% of the airborne methylamine. However, the enzymatic activity of AMO causes formation of formaldehyde. A two-step bioconversion process was therefore proposed. In the first step, airborne methylamine was fed into a CFBR which contained immobilized AMO. In the second step, the gas flow was passed through another CFBR, with alcohol oxidase from the yeast *H. polymorpha* immobilized in CA, in order to decompose the formaldehyde formed in the first step. The proposed system provided almost total elimination of the airborne methylamine and the formaldehyde.

## 1. Introduction

Outdoor and indoor organic air-pollutants pose a serious threat to public health. According to estimates of the World Health Organization (WHO), nearly 800,000 people worldwide die annually from diseases caused by the effects of air-pollution [1]. Methylamine (MA) is the simplest primary amine which is used to produce a variety of important chemical products: pharmaceuticals, pesticides, fuel additives, explosives, solvents, cleaning agents, photographic processing reagents, and chemicals for the tanning of leather and dye processes. MA pollutes the air because of its intensive use in industry and as a result of natural degradation of proteins, amino acids, and other nitrogen-containing compounds in biological samples. Exposure to MA at normal background levels is unlikely to have any negative effects on human health. However, inhalation of air that contains high levels of MA can result in a number of adverse health effects, such as breathing difficulties, a burning sensation, sore throat, headache, and accumulation of fluid in the lungs (pulmonary oedema) [2]. MA can be found in very high concentrations in some kinds of fish, especially of the *Gadoid* species, due to the enzymatic degradation of a natural osmolyte, trimethylamine N-oxide [3, 4].

Recombinant *Saccharomyces cerevisiae* yeast was constructed to overproduce primary amine oxidase (primary amine : oxygen oxidoreductase (deaminating) or amine oxidase, AMO; EC 1.4.3.21), from the thermotolerant yeast *Hansenula polymorpha*. This copper-containing enzyme is known for its ability to decompose primary amines into aldehydes. The following reaction represents a process of MA bioconversion [5, 6]:

(1) $$\begin{array}{c} \text{C}{\text{H}}_{3}\text{N}{\text{H}}_{2}+{\text{H}}_{2}\text{O}+{\text{O}}_{2}\overset{\text{AMO}}{\to }\text{C}{\text{H}}_{2}\text{O}+\text{N}{\text{H}}_{3}+{\text{H}}_{2}{\text{O}}_{2} \end{array}$$

This enzyme has been used for bioanalytical purposes, for example, for spectrophotometric monitoring of the enzymatic decomposition of MA by amine oxidases [7]. To date, this enzyme has not been exploited for bioremediation purposes, that is, for bioconversion of MA into nontoxic products.

The aim of the present work was to construct a recombinant overproducer of AMO from thermostable *H. polymorpha*, isolate and purify the enzyme from the recombinant yeast cells, and design a continuous bioreactor based on immobilized AMO for bioconversion of airborne MA. A two-step continuous fluidized bed bioreactor (CFBR) based on immobilized AMO (step 1) and alcohol oxidase (AOX) (step 2) was designed for bioconversion of airborne MA and the formaldehyde (FA) which is produced in this process. In our previous work we presented a one-step CFBR that performed bioconversion of airborne FA into formic acid by AOX from the yeast *H. polymorpha* that was immobilized in calcium alginate (CA) beads [8].

## 2. Materials and Methods

### 2.1. Chemicals

Paraformaldehyde, MA (40% aqueous solution), PMSF, chromotropic acid, and sodium alginate were purchased from Sigma-Aldrich (Israel).

FA solution (1 M) was prepared by hydrolysis of the corresponding amount of paraformaldehyde in water (300 mg in 10 mL water) by heating the suspension in a sealed ampoule at 105°C for 6 h.

### 2.2. Cloning of the *AMO* Gene into the Expression Vector pYEX-4T-1

**Figure 1:**
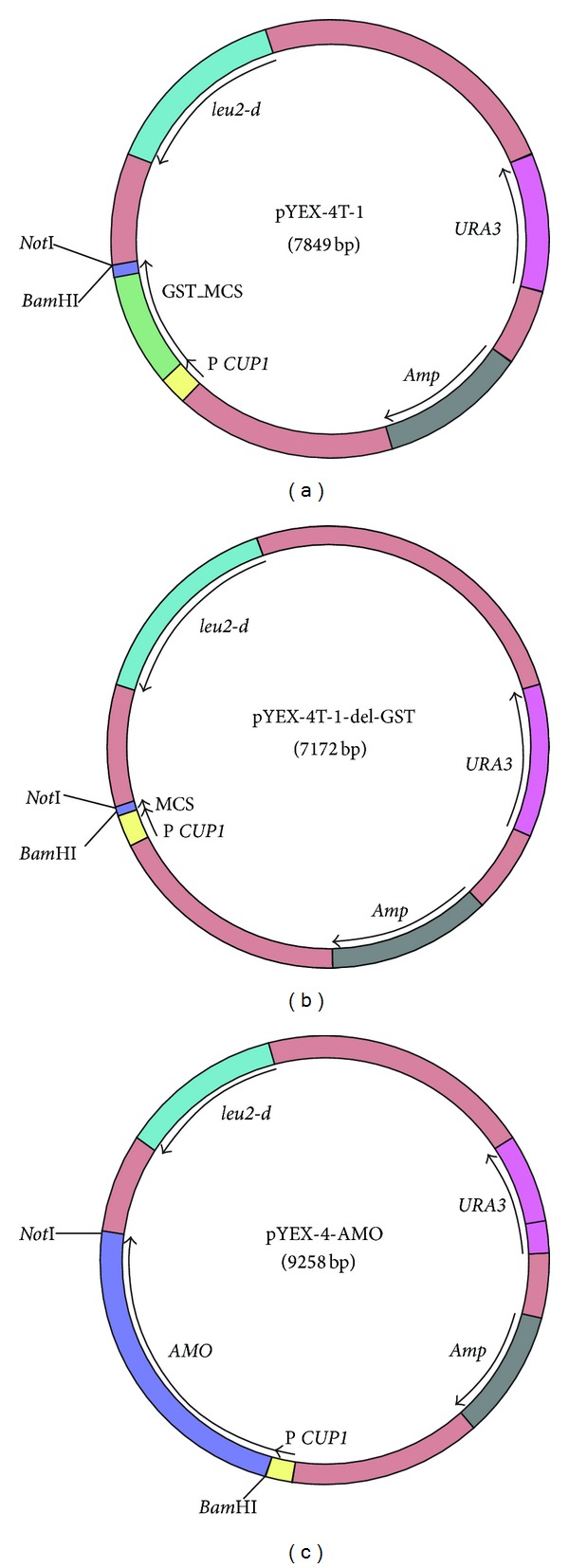
Cloning of the *AMO* gene into the expression vector pYEX-4T-1.

The pYEX-4T-1 plasmid (CLONTECH) was modified prior to cloning of the *AMO* gene. The fragment of the vector containing the *CUP1* promoter and GST was cut out. The sequence encoding the *CUP1* promoter was amplified by PCR and inserted into the plasmid to produce pYEX4T-1-delGST. In order to clone the *H. polymorpha AMO* gene into pYEX4T-1-delGST* Bam*HI and *Not*I restriction sites as well as the sequence encoding for (His)_6_-tag were introduced by amplifying the gene with the primers AMO-for and AMO-rev. The pADES-AMO plasmid was used as a template [6]. The PCR product containing the *AMO* gene was cleaved with *Bam*HI and *Not*I restrictases (Fermentas, Lithuania) and cloned into pYEX-4T-1-del-GST. The resulting plasmid was marked as pYEX-4-AMO (Figure 1), and the correct nucleotide sequence of the AMO reading frame was confirmed by sequencing.

### 2.3. Copper-Inducible Expression of AMO in *Saccharomyces cerevisiae*

Transformants of the yeast strain *S. cerevisiae* C13ABYS86 (*Mat a*, *leu2-3*, *ura3*, *his*, *pra1-1*, *prb1-1*, *prc1-1*, and *cps1-3*) were grown for 18 h at 30°C in minimal synthetic medium containing histidine. The cells were harvested by centrifugation (3,000 ×g, 5 min), resuspended in the same medium, and grown for 2 h to restore log phase growth. Expression was induced by the addition of cupric sulfate to 0.5 mM, and the cultures were maintained for 6–8 h before harvest.

### 2.4. Assay of Enzyme Activities

AMO activity was measured at 30°C and pH 7.0 according to the method described by Haywood and Large [5]. Kinetic studies were carried out under the same conditions within an MA concentration range from 0.02 to 5 mM. The AMO concentration in the incubation mixture was 40 ng mL^−1^. AOX activity was measured as described previously [9]. One unit (1 U) of activity of AMO and AOX was defined as the amount of enzyme which forms 1 *μ*mole of the product per 1 min under standard conditions of the assay. Protein concentration was estimated by the Lowry method. SDS-PAGE was performed in 12% gel according to the Laemmli procedure [10].

### 2.5. Isolation and Purification of AMO

The cells were resuspended in 0.1 M Tris-sulfate buffer, pH 9.4, and treated with 10 mM DTT at 30°C for 10 min. After washing, the cells were suspended in 20 mM potassium phosphate buffer, pH 7.4, containing 1.2 M sorbitol and 2 mg of Zymolyase 20T (Seikagaku Corp., Japan) per 1 g of wet cell weight and incubated at 30°C for 45 min. The spheroplasts were washed once with sorbitol solution and resuspended in 50 mM sodium phosphate buffer, pH 8.0, containing 300 mM NaCl and 2 mM *β*-mercaptoethanol. The spheroplasts were disrupted with glass beads in a BeadBeater (BioSpec Products, USA). After removal of unbroken cells and cell debris by low speed centrifugation, the supernatant was centrifuged at 40,000 ×g for 45 min. The crude extract from 1 L of culture containing 120 mg of protein was applied to a 1 mL Hi-Trap Ni-agarose column (Pharmacia). After removal of nonspecifically bound proteins by washing with 50 mM sodium phosphate buffer, pH 8.0, containing 300 mM NaCl and 2 mM *β*-mercaptoethanol, the (His)_6_-AMO was eluted by a FPLC-mediated 10–500 mM imidazole gradient. The fractions containing high AMO activity were pooled, dialyzed against 50 mM sodium phosphate buffer, pH 8.0, supplemented with 300 mM NaCl and 2 mM *β*-mercaptoethanol, and were used as the final AMO preparation. The specific activity of the pooled preparation was 3.2 U mg^−1^ protein, but in some fractions it reached 13.7 U mg^−1^.

### 2.6. Isolation and Purification of AOX

Cells of the mutant strain *H. polymorpha* C-105 (*gcr1 catX*) overproducing AOX with an additional catalase inactivation mutation [11, 12] were grown in glucose medium [9, 11] and served as a source for AOX isolation. Purification of the enzyme was carried out using a two-step precipitation with ammonium sulfate (at 40 and 60% saturation) in the presence of 1 mM EDTA and 0.4 mM PMSF to inhibit proteases. At 40% saturation, the protein precipitate was discarded, and the AOX precipitate obtained at 60% saturation was collected by centrifugation. The final activity of the enzyme portions used for immobilization was 6–8 U mg^−1^ protein.

### 2.7. Immobilization of AMO and AOX in CA Gel

Aliquots of 1 mL AMO or AOX solutions in 0.05 M PBS, pH 7.5 (buffer A), with 2–40 U mL^−1^ activity, were mixed with 2 mL of 3% (w/v) sodium alginate. The mixtures were dropped into a 2.5 mM CaCl_2_ aqueous solution using a syringe with a 21 G needle under stirring at room temperature and kept for 45 min for beads formation. The obtained gel beads were washed with buffer A.

### 2.8. One-Step Bioconversion of MA in a CFBR

The gel beads with immobilized AMO (8.8–26.6 U g^−1^ of CA) were applied onto a bioreactor of the CFBR type built as a 1 × 30 cm column (1.5 g of beads in 20 mL of buffer A) connected to an air source with a known MA concentration using a scheme analogous to the one described previously by us [8]. The 10–100 ppm MA concentrations in air were obtained by bubbling an air flow of 7–132 mL min⁡^−1^ using a multichannel Ecoline peristaltic pump (Ismatec, Switzerland) through a 0.9–9 mM aqueous MA solution at 25°C. The MA concentration in the gaseous phase at the inlet was calculated, according to Henry's law, taking the Henry constant at 25°C as 1.125 Pa m^3^ mol^−1^ [13]. It was also measured at the inlet and at the outlet by means of adsorption of MA in analytical tubes containing XAD-7 beads covered with 10% 4-chloro-7-nitrobenz-2-oxa-1,3-diazole chloride (SKC Inc., USA) and extraction of MA by tetrahydrofuran (Sigma, Israel) and analyzing the extract by HPLC (Jasco PU 1580, Japan) supplied with a UV/Vis-1578 detector (465 nm) on the Goldsil-100 C18 column (0.46 × 25 cm, 5 *μ*m) according to OSHA Protocol no. 40 [14]. The MA concentration in the aqueous phase was determined by a reaction with lactose and measured by a colorimetric method [15] at 545 nm with UV-Vis Cary-50 (Varian, Australia). The control CFBR column contained CA gel beads without AMO.

### 2.9. Two-Step CFBR System for Continuous Bioconversion of Airborne MA and Produced FA

**Figure 2:**
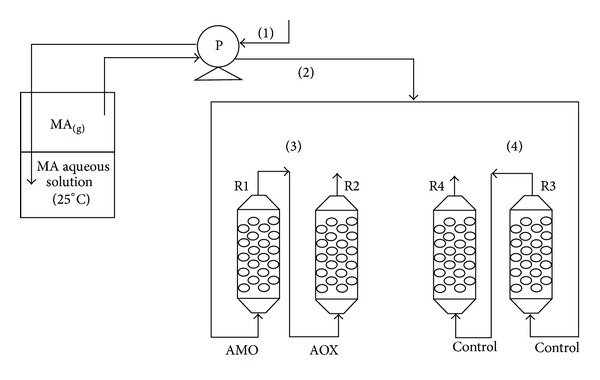
Scheme of a two-step CFBR system for bioconversion of airborne MA by AMO immobilized in CA (R1) and for oxidation of the produced FA by immobilized AOX (R2). The control columns (R3 and R4) contained blank CA-gel beads. (1) an airflow bubbled through aqueous MA solution; (2) a flow of airborne MA; (3) a system of two CFBR in series; (4) a control system and P-a peristaltic pump.

A two-step system consisting of two 1 × 30 cm CFBR columns connected in series was built according to Figure 2. The first reactor, R1, contained gel beads with immobilized AMO (13.3 U g^−1^ of CA) and the second reactor, R2, contained immobilized AOX (6.6 U g^−1^ of CA). Two additional columns (R3 and R4) with blank gel beads served as the control. A flow of airborne MA was split into two streams. The 1st stream was bubbled through R1 and R2, where the outlet from R1 was connected to the inlet of R2. The 2nd stream was bubbled through R3 which was connected to R4. The MA and FA concentrations were tested in the gaseous phase at the outlets of all four reactors, R1–R4, and in the aqueous phase within the reactors. The FA concentration in the gaseous phase was measured with the formaldehyde gas detector (Model FP-40 Riken Keiki, Japan) and in the liquid phase by a standard photometric method using a reaction with 1% chromotropic acid [16].

## 3. Results and Discussion

### 3.1. Cloning of the *AMO* Gene in an Expression Vector

The *AMO* gene, encoding for the microbody matrix enzyme AMO (EC 1.4.3.21) from the yeast *H. polymorpha*, has been cloned, sequenced, and found to contain an open reading frame of 692 amino acids. The enzyme has a dimeric structure with a subunit molecular weight of 78 kDa [17, 18].

The copper-inducible system used for expression of the* AMO* gene was based on the pYEX-4T-1 plasmid, a shuttle expression vector developed for high-level expression of glutathione S-transferase (GST) fusion proteins in the yeast. The copper-inducible *CUP1* promoter from the *S. cerevisiae* metallothionein gene drove the expression of the fusion cassette. pYEX-4T-1 included the *Escherichia coli *Amp^R^ gene, the yeast selectable markers *leu2-d* (a *LEU2* gene with a truncated but functional promoter), and *URA3*.

The *AMO* gene was expressed as a (His)_6_-tagged protein to facilitate its purification. The expression system was based on a modified pYEX-4T-1 vector (see Section 2). The constructed plasmid, designated as pYEX-4-AMO, expressed the (His)_6_-tagged-AMO protein under control of the *CUP1* promoter (Figure 1).

### 3.2. Expression and Purification of Recombinant AMO

**Figure 3:**
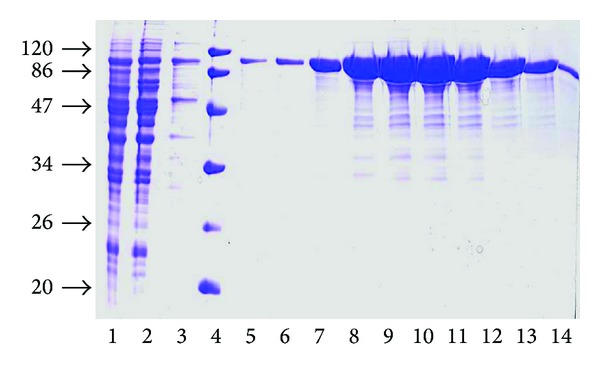
SDS-PAGE in 12% gel of the purified AMO using chromatography on Ni-NTA-agarose. (1) crude extract, 100 *μ*g; (2) flow through the column, 100 *μ*g; (3) washing with 8 mM imidazole, 20 *μ*L; (4) protein molecular weight markers (MW, kDa, 20, 26, 34, 47, 86, and 120; 1.2 *μ*g of each protein); (5)–(14) elution fractions obtained in 10–500 mM imidazole gradient, 20 *μ*L.

Yeast cells carrying the recombinant plasmid were incubated in Cu^2+^-containing selective medium in order to express the *AMO *gene. The active enzyme was accumulated in *S. cerevisiae* cells to a level of 0.7 U mg^−1^ of protein. The highest expression was obtained 6–8 h after induction. The crude extracts were prepared as described in Section 2, and (His)_6_-tagged AMO was purified by Ni-affinity chromatography. The SDS-PAGE results, that illustrate the purification, are presented in Figure 3.

Fractions 8–13 contained high AMO activity and were pooled and used as the final AMO preparation. The enzyme fraction with the highest AMO specific activity of 13.7 U mg^−1  ^was used for kinetic studies.

The specific activity of AMO increased from 4.6 to 20-fold during the course of the purification procedure, and the final yield of AMO protein was 1.6 mg L^−1^ culture. Previous works of others reported a specific activity of 1.1 U mg^−1^ protein, which was determined in the process of MA oxidation by AMO, isolated from the wild type *H. polymorpha *[17] and 5.6 U mg^−1^ protein for the recombinant AMO expressed in *S. cerevisiae *[19]. These data show that the (His)_6_-tagged enzyme which we constructed was at least as active as the recombinant AMO described by Cai and Klinman, without any change in protein structure [19].

The kinetic parameters of the purified (His)_6_-tagged AMO with a specific activity of 13.7 U mg^−1^ protein were studied. The *K*_*m*_ and *V*_max⁡_ (at an enzyme concentration of 40 ng mL^−1^) for MA oxidation at 30°C, pH 7.0, were 0.22 ± 0.011 mM and 0.624 ± 0.050 nmol mL^−1^ min⁡^−1^, respectively, and the *k*_cat_ was 40.0 ± 3.1 s^−1^. For comparison, AMO isolated from the wild type *H. polymorpha* had *K*_*m*_ and *k*_cat_ values of 0.0344 mM and 6.2 s^−1^, respectively, at 25°C and pH 8.0 [20]. The *k*_cat_/*K*_*m*_ ratio for the (His)_6_-tagged enzyme was found to be approximately the same as for the wild type enzyme (182 and 180 mM^−1^ s^−1^, resp.).

It must be emphasized that the recombinant form of the AMO which we constructed differs considerably from the native AMO (without His-tag) previously described in the literature [20]. This AMO contains the (His)_6_-tag attached to the N-terminus which was introduced in order to facilitate its purification by affinity chromatography on Ni-NTA-Sepharose. The correct nucleotide sequence of the AMO-reading frame was confirmed by DNA sequencing, so we assume that changes in kinetic parameters can be explained by the presence of an additional “tail” in the protein structure.

### 3.3. Continuous Bioconversion of Airborne MA

The fundamental possibility for bioconversion of airborne MA was studied using a continuous reactor of the CFBR type that contained CA-gel beads with immobilized AMO, compared to a control system with blank CA-gel beads. An air stream obtained by bubbling various flow rates through aqueous MA solutions with concentrations calculated according to Henry's law was used in order to obtain airborne MA at the desired concentrations [13]. The MA concentration was monitored in the aqueous phase within the reactor and in the gaseous phase at the outlet from the reactor.

**Figure 4:**
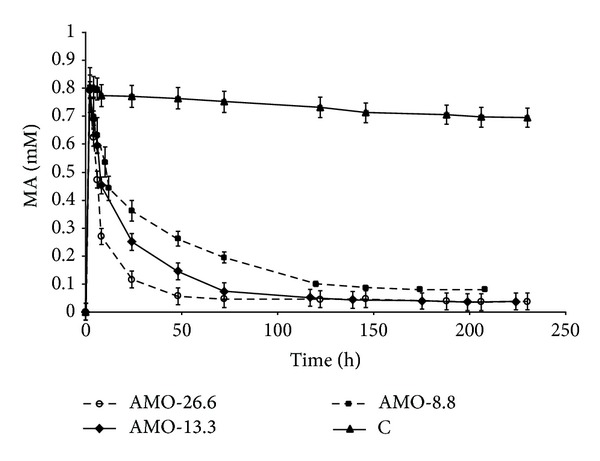
MA concentration in the aqueous phase of the CFBR upon oxidation of MA in the 7 mL min⁡^−1^ air flow with 10 ppm MA inlet concentration by immobilized AMO at various loadings on CA gel: 26.6 U g^−1^ CA, AMO-26.6; 13.3 U g^−1^ CA, AMO-13.3; and 8.8 U g^−1^ CA, AMO-8.8. C (control)—blank beads of CA-gel tested at the same inlet conditions.

In the first stage, effective loading of AMO to the CA-gel was chosen. Bioconversion of MA was tested with three AMO loadings, while the remaining conditions—the 10 ppm inlet MA concentration in the air and airflow of 7 mL min⁡^−1^—were kept constant (Figure 4). This MA concentration was chosen because it lies between the TLV-TWA (5 ppm) and the TLV-STEL (15 ppm) values, where the former corresponds to the level permitted for average chronic exposure based on an 8 h/day and 40 h/week work schedule and the latter to acute exposure to MA for a duration of 15 min that cannot be repeated more than 4 times per day with at least 60 min between exposure periods [21]. The MA concentration in the aqueous phase of the bioreactor increased up to ca. 0.8 mM within the two first hours in all series. In the control experiment (CA without AMO), this MA concentration remained almost unchanged during the 10-day period, with only 10% reduction (Figure 4). The MA concentration in the bioreactors decreased after 2–5 days, depending on the AMO loading, and reached low equilibrium values: 0.035 mM at an AMO loading of 26.6 U g^−1^ CA, 0.06 mM at a loading of 13.3 U g^−1^ CA, and 0.08 mM at a loading of 8.8 U g^−1^ CA (Figure 4). The MA concentration in the gaseous phase at the outlet of the bioreactors did not exceed 0.3–0.5 ppm in all cases, whereas in the control experiment it was 1.3 ppm. The results of this experiment showed that although the best results were obtained with the highest AMO loading, as expected, 13.3 U g^−1^ CA loading of AMO on the gel may be enough for practical purposes, since 96% elimination of airborne MA was achieved. The further experiments were therefore carried out with this loading.

**Figure 5:**
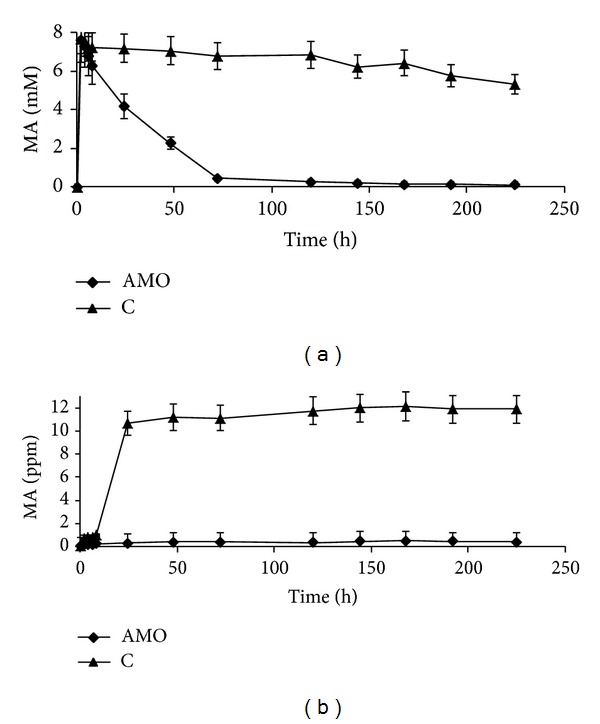
MA concentration in the aqueous (a) and in the gaseous (b) phase of the CFBR upon oxidation of MA in the 7 mL min⁡^−1^ air flow at 100 ppm MA inlet concentration by immobilized AMO at the 13.3 U g^−1^ CA loading. C (control) blank beads of CA-gel, tested at the same inlet conditions.

In the next experiment, the system's ability to bioconvert MA was studied using the 100 ppm MA inlet concentration. When bubbling the airborne MA at this concentration, the concentration of MA in the aqueous phase quickly increased to ca. 8 mM in both the CFBR and the control column, and in the latter case it remained high during the entire experiment. In contradistinction, the MA concentration in the CFBR with immobilized AMO dropped to 0.46 mM after 3 days and decreased further until the end of the experimental period (Figure 5(a)). The MA concentration in the gaseous phase at the outlet of the control column remained at 10–12 ppm, and at the outlet of the bioreactor it did not exceed the safety level of 0.5 ppm during the entire experimental period (Figure 5(b)).

**Figure 6:**
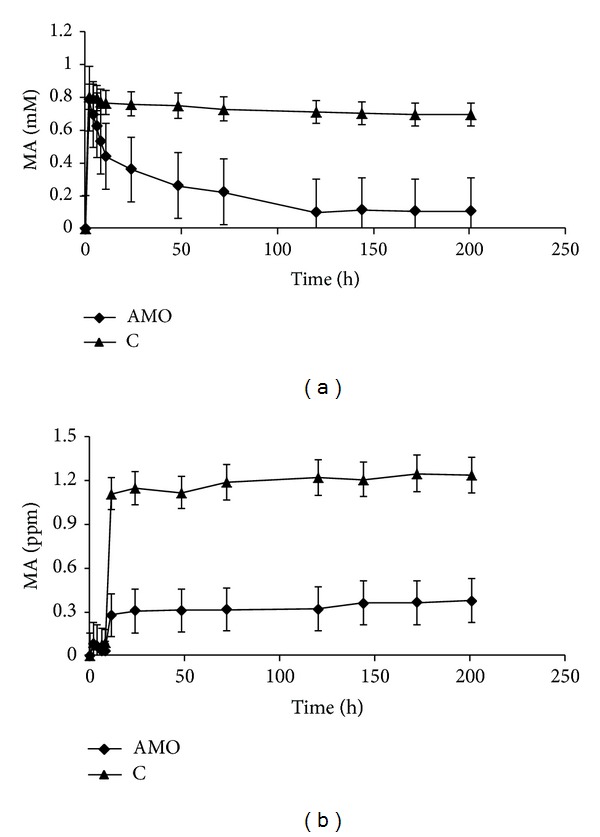
MA concentration in the aqueous (a) and in the gaseous (b) phase of the CFBR upon oxidation of MA in the 132 mL min⁡^−1^ air flow with 10 ppm MA inlet concentration by immobilized AMO at the 13.3 U g^−1^ CA loading. C (control) blank beads of the CA-gel tested at the same inlet conditions.

The CFBR was further examined at a high airflow rate of 132 mL min⁡^−1^ when the AMO loading was 13.3 U g^−1^ CA and the inlet concentration of the airborne MA was 10 ppm (Figure 6). It can be seen that the system functioned at high efficiency in this case as well, and the whole picture was very similar to that obtained with the flow of 7 mL min⁡^−1^ shown in Figure 4 (the AMO-13.3 curve). MA was oxidized to a concentration of less than 0.1 mM in the aqueous phase and to no more than 0.4 ppm at the outlet of the gaseous phase (Figures 6(a) and 6(b), resp.).

### 3.4. Two-Step Continuous Bioconversion of Airborne MA and Produced FA

**Figure 7:**
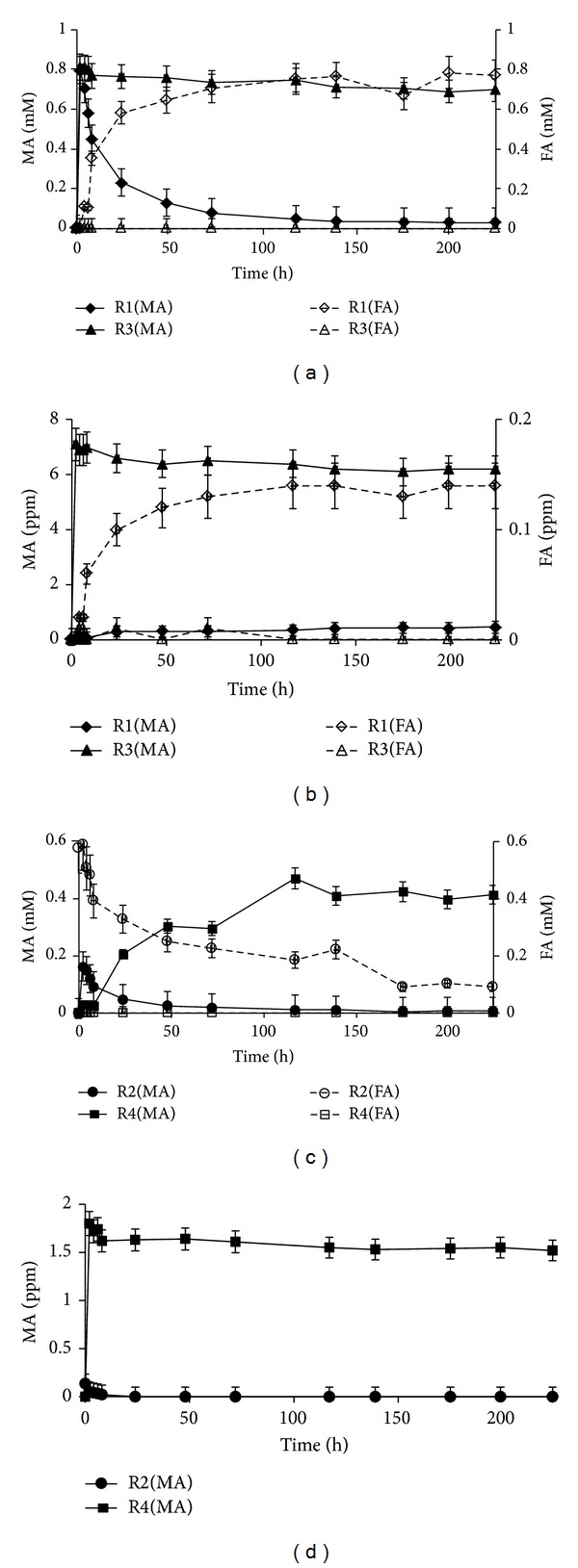
MA and FA concentration in the aqueous ((a) and (c)) and in the gaseous ((b) and (d)) phase of the two-step CFBR system for bioconversion of MA in the 7 mL min⁡^−1^ air flow with 10 ppm MA inlet concentration by immobilized AMO at the 13.3 U g^−1^ CA loading (R1) and for oxidation of the produced FA by immobilized AOX at 6.6 U g^−1^ CA loading (R2). R3 and R4-control reactors connected in series and containing blank beads of CA-gel tested at the same inlet conditions.

Bioconversion of MA is known to produce FA, and, therefore, a system of two continuous CFBR, connected in series, was built for bioconversion of both the airborne MA and the produced FA (Figure 2). The first CFBR (R1) contained CA beads with immobilized AMO (13.3 U g^−1^ CA) and the second (R2) contained beads with immobilized AOX (6.6 U g^−1^ CA). Airborne MA was fed in a continuous regime at the inlet of R1 and the airflow containing the produced FA flowed from the R1 outlet into the R2 inlet. A parallel experiment was performed using the control R3 and R4 reactors which contained blank CA-gel beads. MA and FA were sampled from the aqueous phases of R1–R4 and from the gaseous phase at the outlets from all four columns. The results of the experiment are presented in Figure 7.

The two-step CFBR system demonstrated very high efficiency in eliminating the fed MA and the produced FA (Figure 7). As in the previous experiments, the MA concentration in the aqueous phase first increased to 0.8 mM and then decreased to ca. 0.03 mM in the equilibrium state, while the FA concentration concomitantly increased to 0.8 mM (Figure 7(a), R1(MA) and R1(FA), resp.). This fact indicates that MA was really converted into FA by the enzymatic reaction. In the control experiment, the MA concentration remained at the 0.7–0.8 mM level throughout the experiment and FA was not produced at all (Figure 7(a), R3(MA) and R3(FA), resp.). The MA and FA concentrations in the gaseous phase at the outlet from R1 were 0.4 ppm and 0.14 ppm, respectively (Figure 7(b), R1(MA) and R1(FA)), whereas, in the control experiment, the MA concentration was 6–6.5 ppm and FA was absent (Figure 7(b), R3(MA) and R3(FA)). The MA concentration in the aqueous phase of the R2 reactor was zero, and the FA concentration decreased from 0.6 mM at the beginning of the experiment to 0.09 mM in the equilibrium state (Figure 7(c), R2(MA) and R2(FA)). MA in the control R4 reactor accumulated in the aqueous phase and reached more than 0.4 mM in the equilibrium state (Figure 7(c), R4(MA)). The FA concentration was zero, as expected (Figure 7(c), R4(FA)). The outlet MA concentrations in the gaseous phase were zero and 1.6 ppm for the R2 and R4 reactors, respectively (Figure 7(d), R2(MA) and R4(MA)). The outlet FA concentration was zero in both cases (data not shown). The data presented in Figure 7 clearly show that airborne MA was bioconverted into FA in the R1 reactor and that the produced FA was degraded in the R2 reactor, whereas in the control system MA was not oxidized and, accordingly, FA was not produced at all. Simple trapping of airborne MA by two columns did not lead to its elimination and caused accumulation of MA in the aqueous phase of the R3 and R4 as well as MA exhaust in the gaseous phase.

Several methods have been previously proposed for removal of MA from indoor air. Chou and Shiu[22] reported bioconversion of MA on biofilters arranged in three stages, which contained microorganisms from activated sludge that was obtained from a food-processing wastewater plant. The authors showed that the influent airborne MA, with an input concentration of 100 ppm, was hydrolyzed to ammonia, about a third of the ammonia nitrogen was nitrified to nitrate, and a third was converted into organic or cell nitrogen. Ho's group [23] developed a biofilter based on granular activated carbon coated with immobilized *Paracoccus* sp. CP2 for elimination of 10–250 ppm trimethylamine (TMA), dimethylamine (DMA), and MA. The system effectively treated MA (>93%), DMA (>90%), and TMA (>85%). The coimmobilized *Paracoccus *sp. CP2 and *Arthrobacter* sp. CP1 system was used to achieve complete degradation of TMA and to reduce NH_3_ emission.

Methods for the bioconversion of MA traditionally compete with chemical methods of degradation. Kachina et al. [24] investigated continuous photocatalytic MA oxidation. The authors used titanium dioxide illuminated with UV light at 365 nm as a catalyst. The oxidation process was efficient and the TiO_2_ catalyst showed no deactivation. However, the authors found nitrogen and nitrous oxides among the products of the MA oxidation, in addition to ammonia, which overshadowed the advantages of the method.

Besides destructive methods for the elimination of airborne MA, methods of physical adsorption of airborne MA on several carbon materials were also studied [25]. Despite a high adsorption efficiency, physical adsorption does not lead to degradation of MA and is therefore less preferable than chemical or biological conversion methods.

The two-step AMO/AOX enzyme system presented in this study enabled the achievement of complete bioconversion of MA and of FA. It also enabled the neutralization of the ammonia by-product of the first process by the formic acid by-product of the second process, since, according to the stoichiometry of the bioconversion processes, these by-products are produced in equal concentrations.

## 4. Conclusions

A recombinant *S. cerevisiae* yeast overproducing AMO from the thermotolerant yeast *H. polymorpha* was constructed. The novel (His)_6_-tagged enzyme was purified from the cell-free extract of the recombinant strain by metal-affinity chromatography and characterized electrophoretically and kinetically. The enzyme that was immobilized in CA beads demonstrated a high capability for eliminating airborne MA in a continuous regime, when employed in a two-step CFBR designed for bioconversion of airborne MA and the produced FA.
